# Anticancer Activity of *Combretum fragrans* F. Hoffm on Glioblastoma and Prostate Cancer Cell Lines

**DOI:** 10.31557/APJCP.2021.22.4.1087

**Published:** 2021-04

**Authors:** Isaac Silvère Gade, Tagne Simo Richard, Corinne Chadeneau, Paule Seite, Brigitte Vannier, Alex De Theodore Atchade, Paul F. Seke Etet, Emmanuel Talla, Armel H. Nwabo Kamdje, Jean-Marc Muller

**Affiliations:** 1 *Department of Organic Chemistry, Faculty of Science, University of Yaoundé I, Yaoundé, Cameroon. *; 2 *UFR Sciences Fondamentales et Appliquées, Team “Récepteurs, Régulations, Cellules Tumorales” (2RCT)-EA 3842 CAPTuR, Pôle Biologie Santé-Bât. B36/B37, University of Poitiers, 1 rue Georges Bonnet-TSA, France. *; 3 *Department of Chemistry, Faculty of Science, University of Ngaoundere, Ngaoundéré, Cameroon. *; 4 *Department of Biomedical Sciences, Faculty of Science, University of Ngaoundere, Ngaoundéré, Cameroon. *; 5 *Department of Physiological Sciences and Biochemistry, FMBS, University of Ngaoundere, Garoua, Cameroon. *

**Keywords:** Combretum fragrans, cancer cells, apoptosis, ERK1/2, Akt

## Abstract

**Background:**

Cancer incidence has been growing in an alarming rate worldwide and new therapeutics are needed, particularly for intractable and chemoresistant cases. We evaluated the cytotoxic effects of *Combretum fragrans* F. Hoffm (Combretaceae) on glioblastoma (U87MG and C6) and prostate (PC-3) cancer cell lines.

**Methods:**

The cytotoxic effect of the methanolic extract of the stem bark of *Combretum fragrans* was assessed using XTT (2,3-bis (2-methoxy-4-nitro-5-sulfophenyl)-5-[(phenylamino) carbonyl]-2H-tetrazolium hydroxide) test. Expressions of Akt and ERK1/2 were determined using Western blot technique, while Caspase-3/7 kits were used to evaluate caspase-3/7 activity.

**Results:**

*C. fragrans* extract inhibited the proliferation of U87 (IC_50_ = 20.13 µg/mL), C6 (IC50 = 12.17 µg/mL), and PC-3 (IC50 = 11.50 µg/mL) cells. Treatment with the extract resulted in lower levels (p < 0.001) of phospho-ERK1/2 and phospho-Akt in U87 cells, and instead, higher levels of phospho-ERK1/2 (p < 0.001) in C6 and PC-3 cells. An increase in caspase-3/7 activity was observed, mainly after 24 hours of treatment, indicating the activation of apoptotic processes.

**Conclusion:**

Altogether, these results suggest that *C. fragrans* have potent anticancer properties. This plant should be further investigated for developing new anticancer drugs.

## Introduction

Modern lifestyle exposes us to various factors that may contribute to the activation of oncogenes (Djiogue et al., 2013; Kipanyula et al., 2013; Makarem et al., 2017; Nair-Shalliker et al., 2017), which in turn may induce disturbances in cell growth, differentiation, and apoptosis, resulting in cancers (Seke et al., 2012; Nwabo et al., 2017). Cancers are pathologies mainly resulting from both the inability of cells to control their divisions and by the loss of mechanisms of programmed cell death. Notably, they are a leading cause of death worldwide and there are continuous increases in the incidence and mortality rates. In 2018, the global cancer burden was estimated at 18.1 million new cases and 9.6 million deaths. It is estimated that there will be 21 million new cancer cases and 17 million cancer deaths per year by 2030 (Siegel et al., 2016). 

Developing drug-resistant cancer in patients is a major obstacle in both conventional chemotherapeutics and novel targeted therapeutics (Butler et al., 2013). About 90% patients obtain chemotherapeutic failure due to generation of drug-resistant cancer cells, even in their initial treatment. The major mechanisms of drug-resistance in cancer cells are diversified and complicated processes, including activating of DNA repair, decreasing drug influx, confiscating of drugs within intracellular organelles, increasing drug efflux, disabling of apoptosis pathways, and triggering of immune response etc (Luqmani, 2005). Moreover, chemotherapy agents, the first line conventional anticancer drugs, are associated with adverse effects and severe adverse effects in patients, including nausea and vomiting, alopecia, marked affections of erythropoietic and immune functions, etc. Chemotherapy agents are also expensive and not easily accessible in developing countries (Singh et al., 2018). Hence, new anticancer agents are needed in the field. 

Natural products are recognized as a promising source of bioactive compounds with a high potential for development as new preventive and therapeutic anticancer agents (Xu et al., 2014). About 60 % of drugs currently used for treating cancer were isolated from natural products (Yin et al., 2019), particularly medicinal plants, which are even commonly used as alternative anticancer therapies. Not surprisingly, several medicinal plants showed interesting anticancer activities, such as *Annona muricata*, *Ailanthus altissima*, *Tabernaemontana elegans*, and *Urtica membranacea* (Bandgar et al., 2010; Solowey et al., 2014; Apriyanto et al., 2018) , and various anticancer drugs commonly used were isolated from plants like *Catharanthus roseus* G. Don., *Taxus brevifolia* Nutt., *Camptotheca acuminata* Decne, *Combretum caffrum* and *Podophyllum peltatum* L. (Cragg and Pezzuto, 2016).

*Combretum fragrans* F. Hoffm is a medicinal plant of the Combretaceae family, which is widely use in African traditional medicine to treat various diseases and conditions, including pain and inflammation, cough, hypertension, wounds, syphilis, leprosy, fungal infections of the scalp, malaria, gonorrhea, and snake bite (Maregesi et al., 2007; Maima et al., 2009; Mbiantcha et al., 2018). In addition, in the Northern part of Cameroon, this plant is also used to treat jaundice, diabetic foot wounds, ulcers, and cancers. Previous pharmacological studies of *C. fragrans* extracts reported antibacterial, antifungal and antiproliferative properties (Fyhrquist et al., 2006; Maregesi et al., 2008). However, data on the apoptotic potential and the mode of action of these extracts are lacking.

In the present study, we assessed the anticancer activity and mode of action of the methanolic extract of the stem bark of *C. fragrans* in glioblastoma and prostate cancer cell lines.

## Materials and Methods

*Plant material and extract preparation*

The stem bark of *C. fragrans* was collected in May 2017 from the village of Padarmé, North Region, Cameroon. A voucher specimen N° 39753/HNC was deposited at the National Herbarium of Cameroon (NHC). A total of 1 kg of shade-dried and powdered stem bark of *C. fragrans* was macerated in 5 L of methanol for 2 days with intermittent stirring. The solution obtained was filtered, and the solvent was removed. The procedure was repeated three times to obtain a total of 40 g of the methanolic extract of the stem bark of *C. fragrans* (extraction yield: 0.04 %).

*Cell lines and cell culture*

Cells of the human U87MG and rat C6 glioblastoma lines were grown in Dulbecco’s modified Eagle’s medium (DMEM, 1 g/L glucose) containing GlutaMAX^TM ^(Gibco) and sodium pyruvate (Invitrogen), supplemented with 10% of fetal calf serum, 100 U/ml penicillin and 100 µg/ml streptomycin (Gibco). Cells of PC-3 human prostate cancer line were grown in the same conditions, except for the use of DMEM medium with 4.5 g/L glucose. The cells were maintained at 37°C in a humidified incubator (95 % air and 5 % CO_2_).

*XTT cytotoxicity assay*

The U87 and C6 glioblastoma (GBM) cells, as well as the PC-3 prostate cancer cells were plated in 96-wells plate respectively at a density of 5×10^3^, 3×10^3^ and 4×10^3^ cells/well in 100 µL of corresponding medium. Then, cells were allowed to attach overnight in growth medium. After a 24h incubation, the medium was replaced with fresh medium containing different concentrations of the tested extracts (3.15 - 200 µg/mL). Control cells were treated with DMSO (0.5%) used to dissolve samples. After 72h of treatment, cell viability was measured using the Cell Proliferation Kit II (XTT) (Roche, Mannheim, Gremany) as recommended by the manufacturer. Briefly, 50 µL of XTT (2,3-bis[2-Methoxy-4-nitro-5-sulfophenyl]-2Htetrazolium-5-carboxyanilide inner salt) labeling reagent mixture solution was added in each well, and then, the plate was incubated for 4h. XTT reagent is converted by the metabolically active cells into an orange formazan dye, and the formazan formed is directly proportional to the living cells. The absorbance was measured at 490 nm using a spectrophotometric microplate reader.

*Total protein extraction*

To study the effect of the extract of *C. fragrans* on the level of ERK1/2 and Akt proteins expression, the U87, C6 and PC-3 cancer cells were seeded in Petri dishes and allowed to grow up to 90 % confluence. Then, the medium was removed and cells were treated with the medium containing the extract at 20 and 40 µg/mL for 1h, 6h and 24h. Control cells were treated with the medium containing the solution used to dissolve the extract (0.5 % DMSO). After treatment, the medium was removed, and cells were washed twice with cold phosphate-buffered saline (PBS). Then, cells were scrapped into ice-cold lysis buffer (10 mM Tris-HCl, pH 7.5; 0.5 mM EDTA, pH 8; 0.5 % CHAPS and 10 % glycerol) supplemented with protease and phosphatase cocktail inhibitors (Pierce 88669, 40X). After 30 min on ice, lysates were centrifuged at 4°C for 20 min at 13,000g. The supernatant was collected and stored at - 80°C. Protein concentration was determined using the DC Protein Assay (Bio-Rad, Hercules, CA, USA).

*Western blot*

Proteins (20 µg) were resolved in 10% SDS–PAGE and transferred on polyvynilidene fluoride (PVDF) membranes. Membranes were blocked using 5% nonfat milk in tris-buffered saline (TBS) containing 0.1% Tween 20 (Sigma–Aldrich) (TBST) for 1 h at room temperature and then incubated overnight at 4°C with the following primary antibodies diluted in blocking solution: monoclonal rabbit anti-phospho-Akt (Ser473) (1:1,000) or anti-Akt (pan) (1:1,000) (Cell Signaling), monoclonal rabbit anti-phospho-p44/42 MAPK (ERK 1/2) (Thr202/Tyr204) (1:1,000) or anti-p44/42 MAPK (ERK 1/2) (1:1,000) (Cell Signaling), monoclonal mouse anti-GAPDH antibody (1:80,000) (Abcam). After 3 washes of 10 min with TBST, chemiluminescent signals were generated using Millipore Luminata forte (Merck, Darmstadt, Germany) and images were captured with PXi system (Syngene International Ltd, Bangalore, India). The blots were quantified by densitometry using Image J software (NIH, Bethesda, MD, USA).

*Assay for apoptosis*

Apoptosis was detected in cells by evaluating the caspase-3/7 activity and cleaved poly(ADP-ribose)polymerase 1 (PARP-1) expression. Cells were treated with *C. fragrans* extract at 20 and 40 µg/mL for 1h, 6h and 24h. The caspase-3/7 enzymatic activity was measured using the Apo-ONE^®^ Homogeneous Caspase-3/7 kits assay (Promega) according to the manufacturer’s recommendations. The assay is based on the cleavage of non-fluorescent caspase substrate by caspase-3/7 that creates a fluorescent product; the amount of fluorescent product generated is proportional to the amount of caspase-3/7 cleavage activity present in the sample. The intensity of the emitted fluorescence was measured at 485 nm using a spectrophotometric microplate reader (Mithras, Berthold Technologies, Bad Wildbad, Germany). 

*Statistical analysis*

The results were expressed as mean ± standard error of the mean (SEM). IC_50_ values were calculated from the sigmoidal nonlinear regression curve. Statistical comparisons were performed with the Friedman test using GraphPad Prism. The statistical significance was set at P < 0.05.

## Results

*In Vitro cytotoxic effects of the extract of C. fragrans*

The investigation of the cytotoxic potential of the methanolic extract of *C. fragrans* was conducted on three cancer cell lines (U87MG and C6 and PC-3). The extract exhibited a strong inhibition of the proliferation of U87, C6, and PC-3 cells, with similar IC_50_ values in PC-3 (11.50 µg/mL) and C6 (12.17 µg/mL) cells, and an almost twice higher IC_50_ value in U87 (20.13 µg/mL) cells. 

*Relative activities of ERK and Akt*

**Figure 1:**
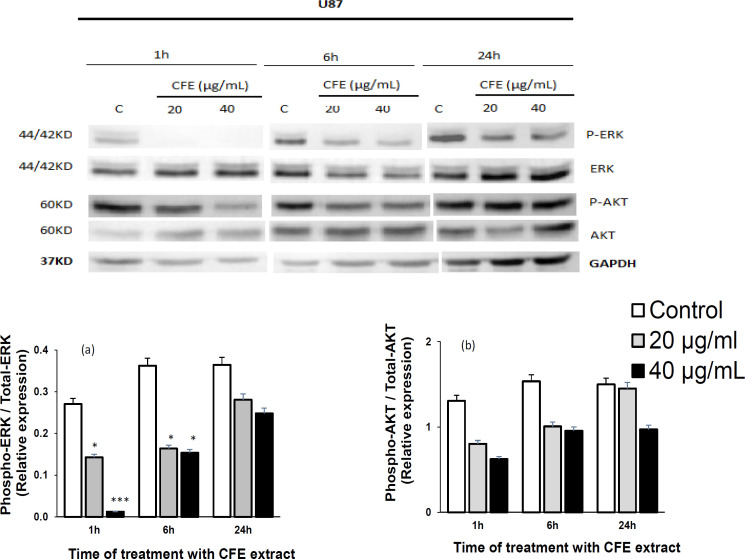
Effects of *C. fragrans* Treatment on ERK and Akt Activities in U87 Cells. Representative experiment showing western immunoblot expressions of ERK and Akt (a) and quantification of active phospho-ERK / total ERK and phospho-Akt / total Akt (b). Cells were treated for 1h, 6h and 24h with *C. fragrans* extract (CFE). Results are from three independent experiments performed in duplicate. Friedman test: * p < 0.05 and *** p < 0.001. C: control cells

**Figure 2:**
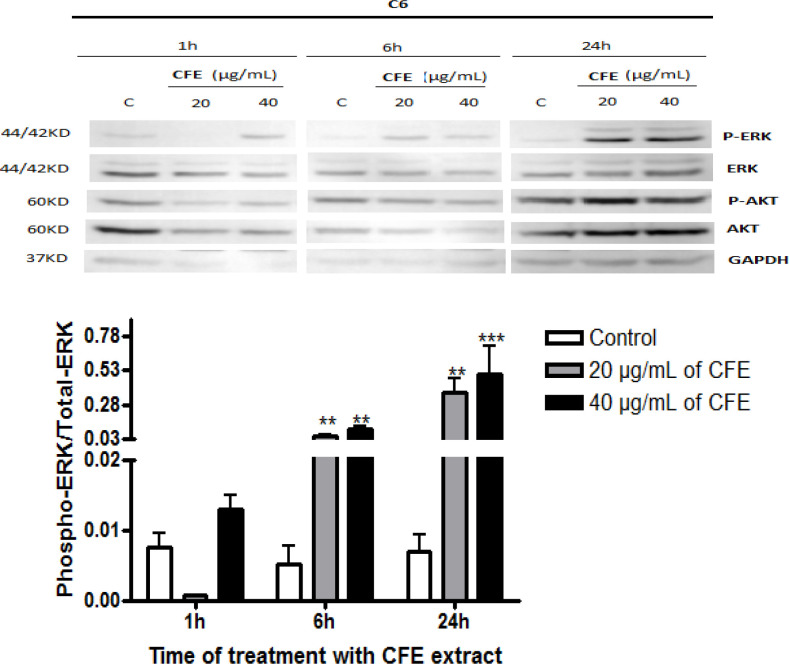
Effects of *C. fragrans* Treatment on ERK and Akt Activities in C6 Cells. Representative experiment showing Western immunoblot expressions of ERK and Akt and quantification of active phospho-ERK / total ERK. Cells were treated for 1h, 6h and 24h with *C. fragrans* extract (CFE). Results are from three independent experiments performed in duplicate. Friedman test: ** p < 0.01 and *** p < 0.001. C, control cells

**Figure 3:**
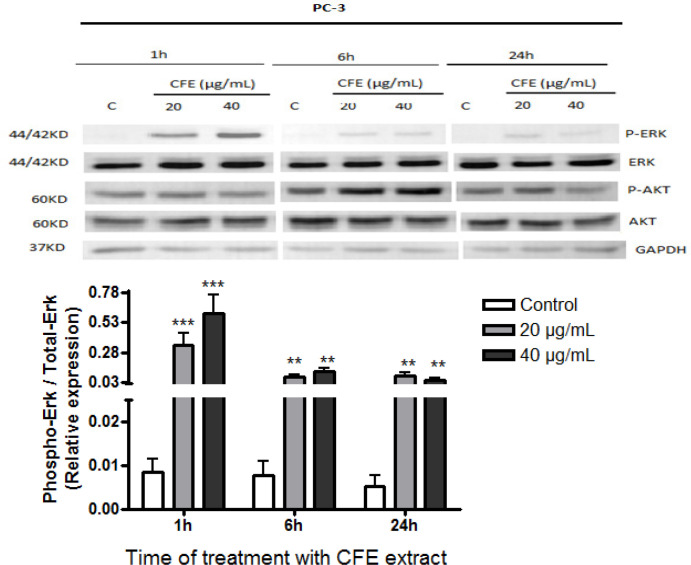
Effects of *C. fragrans* Treatment on ERK and Akt Activities in PC-3 Cells. Representative experiment showing Western immunoblot expressions of ERK and Akt and quantification of active phospho-ERK / total ERK. Cells were treated for 1h, 6h and 24h with *C. fragrans* extract (CFE). Results are from three independent experiments performed in duplicate. Friedman test: ** p < 0.01 and *** p < 0.001. C, control cells

Figures 1, 2, and 3 show the effects of *C. fragrans* treatment for 1h, 6h and 24h on ERK and Akt expressions in U87, C6, and PC-3 cells, respectively. Relative activities of ERK (ratio phospho-ERK / total ERK) (Figure 1a) and Akt (phospho-Akt / total Akt) (Figure 1b) declined significantly compared to control cells (p < 0.05) in U87 glioblastoma cells, particularly after 1h of treatment (Figure 1). Instead, *C. fragrans* extract induced strong and sustained activations of ERK1/2 in C6 glioblastoma cells and PC-3 prostate cancer cells, particularly strong after 24h of treatment and 1h of treatment, respectively, compared to control cells (p < 0.01) (Figures 2 and 3). No marked change in Akt activity was observed in either C6 or PC-3 cells (data not shown).

*Caspase-3/7 activity*

**Figure 4:**
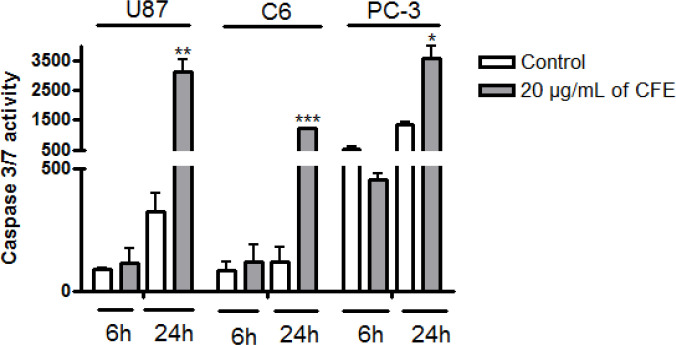
Effects of *C. fragrans* Extract on Caspase-3/7 Activity in U87, C6 and PC-3 Cells. Cells were treated with 20 µg/mL of C. fragrans extract (CFE) for 6 and 24h. Results were obtained from two independent experiments performed in triplicate. Friedman test: * p < 0.05; ** p < 0.01 and *** p < 0.001

Figure 4 shows the effects of *C. fragrans* treatment for 6h and 24h on caspase-3/7 activity in U87, C6 and PC-3 cells. *C. fragrans* extract strongly increased the caspase-3/7 activity in all the cancer cell lines tested, particularly after 24h of treatment (Figure 4). 

*PARP-1 expression*

**Figure 5:**
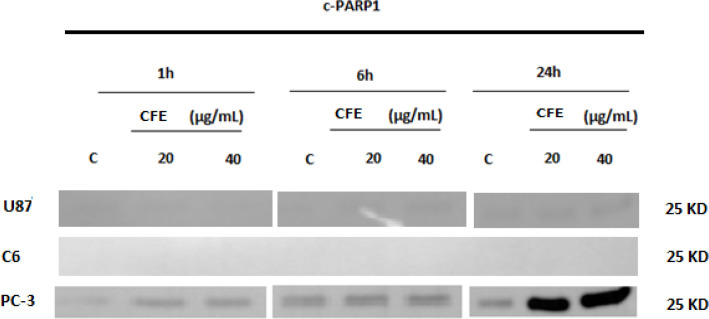
Effects of *C. fragrans* Treatment on *PARP 1* Expression. Representative experiment showing Western immunoblot expressions of cleaved PARP 1 in U87, C6 and PC-3 cells following 1, 6 and 24h of treatment with *C. fragrans* extract (**CFE**) at 20 and 40 µg/mL. **C**, control cells

Figure 5 shows the effects of *C. fragrans* extract on cleaved PARP 1 expression in U87, C6 and PC-3 cells following 1, 6 and 24h of treatment. A slight increase in the expression of cleaved PARP-1 was detected in U87 cells after 24h of treatment with *C. fragrans*, while cleaved PARP-1 was not detected in C6 cells even after 24h of treatment with *C. fragrans* (Figure 5). On the other hand, slight increases in the expression of cleaved PARP-1 in PC-3, which were observed from 1h of treatment with *C. fragrans*, became marked after 24h of treatment (Figure 5).

## Discussion

The results of the present study suggest that *C. fragrans* extract mediated a strong inhibition of the proliferation of U87, C6, and PC-3 cells, possibly through ERK and Akt signaling modulation. The extract induced a significant decrease in relative activities of ERK (ratio phospho-ERK / total ERK) and Akt (phospho-Akt / total Akt) compared to control cells in U87 glioblastoma cells, particularly after 6h of treatment. ERK 1/2 and Akt are key components of MAP kinase and PI3K signaling pathways, respectively. This finding indicates that the extract probably induced apoptosis in U87 through the modulation of ERK and Akt signaling, and is consistent with previous reports on effects of other medicinal plants (Wang et al., 2004; Yu et al., 2017). These signaling pathways are key players in cancer pathogenesis due to their involvement in malignant cell proliferation, differentiation and survival ( Coffer et al., 1998; Cobb, 1999; Xiao and Singh, 2002), including in glioblastoma (Molina et al., 2010) and prostate cancer (Albrecht et al., 2008). 

Interestingly, on the other hand, *C. fragrans* extract induced a sustained and strong activation of ERK in C6 and PC-3 cells without changes in Akt expression. Considering that early studies show that prolonged ERK activation causes cell growth arrest and cell death (Wang et al., 2000; Ballif and Blenis, 2001), it appears that *C. fragrans* extract may induce apoptosis through strong and prolonged activation of ERK1/2. It is now established that although ERK is a pro-survival factor in the MAP kinase family and contributes to the regulation of cell proliferation and differentiation, under some circumstances like prolonged activation, ERK1/2 can induce cell cycle arrest (Marshall, 1995; York et al., 1998) and apoptosis ( Xiao and Singh, 2002; Rangaswami et al., 2006; Lin et al., 2007). ERK1/2 prolonged activation is though to be present in approximately 30% of tumors (Albrecht et al., 2008). 

In conclusion, altogether, the results of the present study indicate that the methanolic extract of the stem bark of *C. fragrans* strongly inhibited the growth of U87 human glioblastoma cells, C6 rat glioblastoma cells and PC-3 prostate cancer cells. The extract induced apoptosis which is correlated with a significant inhibition of ERK1/2 and Akt activities in U87 cells, and a strong and sustained activation of ERK1/2 in C6 and PC-3 cells. Therefore, *C. fragrans* is a promising source of useful molecules for anticancer therapy.

## Author Contribution Statement

The authors confirm contribution to the paper as follows: study conception and design: Richard Tagne Simo, Armel H. Nwabo Kamdje, Emmanuel Talla, Jean-Marc Muller; data collection: Isaac Silvère Gade, Corinne Chadeneau, Paule Seite, Brigitte Vannier; analysis and interpretation of results: Isaac Silvère Gade, Corinne Chadeneau, Alex De Théodore Atchade; draft manuscript preparation: Isaac Silvère Gade, Richard Tagne Simo, Paul F. Seket Etet. All authors reviewed the results and approved the final version of the manuscript.
